# Effect of alendronate sodium on the expression of mesenchymal-epithelial transition markers in mice with liver fibrosis

**DOI:** 10.3892/etm.2012.759

**Published:** 2012-10-22

**Authors:** WAN-RONG BI, CAI-XIA JIN, GUO-TONG XU, CHANG-QING YANG

**Affiliations:** 1 Department of Gastroenterology and Digestive Disease Institute, Tongji Hospital Branch;; 2 Departments of Stem Cells Laboratory and; 3 Gastroenterology, Tongji Hospital, Tongji University, Shanghai, P.R. China

**Keywords:** alendronate sodium, transforming growth factor-β1, bone morphogenetic protein-7, epithelial mesenchymal transition, liver fibrosis

## Abstract

The aim of this study was to explore whether alendronate sodium regulates tissue remodeling by controlling the transforming growth factor (TGF)-β1-induced epithelial-mesenchymal transition (EMT) and bone morphogenetic protein (BMP)-7-induced mesenchymal-epithelial transition (MET) in CCl_4_-induced hepatic fibrosis in mice. A mouse model of CCl_4_-induced hepatic fibrosis was evaluated using the hematoxylin and eosin (HE) and Masson’s trichrome staining histological methods. The activities of serum alanine aminotransferase (ALT) and aspartate aminotransferase (AST) were measured using an automated biochemical analyzer. The expression of TGF-β1, α-smooth muscle actin (α-SMA), BMP-7 and E-cadherin in the hepatic tissue was detected using immunohistochemistry. The mRNA and protein levels of TGF-β1, α-SMA, BMP-7, fibroblast-specific protein 1 (FSP1), E-cadherin and N-cadherin were detected using RT-PCR and western blot analysis. Immunohistochemical and molecular biochemical examination revealed that alendronate sodium significantly arrested the progression of hepatic fibrosis. Alendronate sodium caused significant amelioration of liver injury and reduced the activities of serum ALT and AST (P<0.001). Furthermore, alendronate sodium markedly reduced TGF-β1 and α-SMA mRNA expression and increased BMP-7 and E-cadherin in the mouse liver tissue (P<0.001). Alendronate sodium significantly arrested the progression of hepatic fibrosis. The underlying mechanism was associated with changes in the redox state, which remains variable in liver fibrosis, and depends on the balance between TGF-β/smad- and BMP-7*-*modulated mechanisms which regulate EMT and MET in multifunctional progenitors.

## Introduction

The pathogenesis of liver cirrhosis is complex, involving several signal transduction pathways and several liver cell types (1). Stellate cells, non*-*parecnhymal cells in the liver, become activated during liver injury and initiate programs of transforming growth factor (TGF)-β1 production and extracellular matrix (ECM) remodeling that contribute to the apoptosis of hepatocytes (2). Ethanol injury leads to apoptosis of hepatocytes and an increase in the production of fibrogenic cytokines, including TGF-β1. TGF-β1 has also been revealed to induce de-differentiation and epithelial-mesenchymal transition (EMT) in hepatocytes (3).

The bone morphogenetic proteins (BMPs) are a large class of multifunctional growth factors and are involved in a major developmental signaling pathway critical for embryogenesis and tissue generation in organs, including the kidney and lung (4). However, BMPs are also essential during postnatal life and regulate cell proliferation, differentiation, apoptosis, angiogenesis and the secretion of ECM components (5). BMP-7 is considered to have inhibitory effects since it is able to counteract TGF-β1-induced fibrotic effects *in vitro* and reverse established fibrosis in organs as diverse as the kidney, heart and colon (6).

Alendronate sodium is a bisphosphonate that acts as a specific inhibitor of osteoclast-mediated bone resorption. Bisphosphonates are synthetic analogs of pyrophosphate which bind to hydroxyapatite in bone. Alendronate sodium is chemically described as (4-amino-1-hydroxybutylidene) bisphosphonic acid monosodium salt trihydrate. Alendronate sodium is a white, crystalline, non-hygroscopic powder (7).

The present study demonstrates for the first time that alendronate sodium significantly arrests the progression of hepatic fibrosis. The underlying mechanism was associated with changes in the redox state and involved marked decreases in the expression of TGF-β1 and α-smooth muscle actin (α-SMA) and upregulation of the expression of BMP-7 and E-cadherin in liver tissue.

## Materials and methods

### Chemicals and materials

Glass slides (75×25 mm^2^) were obtained from Gibco (Carlsbad, CA, USA) and (3-acryloxypropyl) trichlorosilane was purchased from Gelest, Inc. (Morrisville, PA, USA). Streptavidin-conjugated Alexa 546, AlexaFluor 488 anti-mouse IgG, BMP-7 and TGF-β1 were obtained from Sigma-Aldrich (St. Louis, MO, USA). Mouse anti-E-cadherin antibody was purchased from BD Biosciences (Franklin Lakes, NJ, USA). Concentrated phosphate-buffered saline (10X PBS) was purchased from Lonza (Shanghai, China). Minimal essential medium (MEM), sodium pyruvate, nonessential amino acids, fetal bovine serum (FBS), Superscript III, RNaseOut (RNase inhibitor) and dNTPs were purchased from Invitrogen (Shanghai, China). Polypropylene microarray plates (384 well) were obtained from Genetix (Shanghai, China). Goat anti-mouse cross-adsorbed albumin antibody was obtained from Sigma-Aldrich. Formalin was purchased from Fisher (Shanghai, China). The ApopTag Red *in situ* apoptosis detection kit was obtained from Chemicon (Shanghai, China). DAPI stain mounting media were purchased from Vectorshield (Shanghai, China).

### Animals

Adult gender-matched (n=20 each gender) C57BL mice weighing 200±10.2 g were purchased from Tongji University Laboratories (Shanghai, China) and fed on a commercial diet with water. All animal experiments were performed according to the National Institute of Health (NIH) guidelines for the ethical care and use of laboratory animals and the study was approved by the Tongji Animal Care and Use Committee of China.

### Drugs

The alendronate sodium tablets (10 mg) also contained carnauba wax. Each bottle of the oral solution contained 91.35 mg alendronate monosodium salt trihydrate, which was the molar equivalent of 70 mg free acid.

### Groups

Adult numbered mice (n=40) were assigned randomly to one of four groups. In the normal control group, 10 mice received intraperitoneal injections of olive oil (0.5 ml/100 mg) twice each week. In the alendronate sodium control group, 10 mice received intraperitoneal injections of olive oil (0.5 ml/100 mg) and alendronate sodium (25 mg/kg) at the same time, twice each week. In the liver fibrosis model group, 10 liver fibrosis model mice received intraperitoneal injections of 40% CCl_4_ and olive oil mixture (0.5 ml/100 mg, Sigma-Aldrich) as previously described (8). In the alendronate sodium-treated group, 10 mice received intraperitoneal injections of 40% CCl_4_ and olive oil admixture (0.5 ml/100 mg) twice each week, as well as alendronate sodium (25 mg/kg) at the same time. The mice were sacrificed after 8 weeks of treatment.

### Cell isolation and culture

Liver epithelial cells were isolated from normal Sprague-Dawley mice as follows: following *in situ* perfusion of the liver with pronase (Roche, Indianapolis, IN, USA) and collagenase (Roche), dispersed cell suspensions were layered in a discontinuous density gradient of 5.8% Larcoll (Sigma-Aldrich) and 15.6% Histodenz (Sigma-Aldrich). The resulting upper layer consisted of >98% liver epithelial cells. The purity and viability were verified by phase-contrast microscopy with examination of autofluorescence and propidium iodide exclusion (50 *μ*g/ml; Roche). Liver epithelial cells were cultured in 10% serum-supplemented DMEM (Invitrogen) with streptomycin-penicillin.

### Reverse transcription and real-time quantitative PCR (RT-PCR) analysis

PBMC solution (100 *μ*l) priority was added to 300 *μ*l Trizol lysate and 100 *μ*l chloroform at 4°C, and spun at 1,3200 rpm for 15 min. The liquid supernatant affiliated isopyknic avantin, −20°C deposit 30 min, 4°C 1,3200 rpm for 15 min. The liquid supernatant was added to 300 *μ*l 75% alcohol at 4°C and spun at 1,3200 rpm for 15 min. The liquid supernatant was added to diethyl carbonate (DEPC). This solution was used as the total RNA solution of PBMC. Total RNA solution (10 *μ*l) with 1 *μ*l random primers (0.2 *μ*g/*μ*l) and 1 *μ*l DEPC was denatured at 70°C for 5 min, then mixed with 4 *μ*l 5X RT buffer, 1 *μ*l RNasin (20 *μ*g/*μ*l), 2 *μ*l 10 mM dNTP mix and 1 *μ*l reverse transcription enzyme M-MLV (20 *μ*g/*μ*l) at 42°C for 60 min. Next, the solution was heated to 70°C for 10 min, then cooled in ice water and stored at −20°C. The RT-PCR used a 20-*μ*l reaction system with 2 *μ*l RNase inhibitor, 7.2 *μ*l deionized water, 10 *μ*mol/l forward and reverse primers (0.4 *μ*l each) and 10 *μ*l 2X RT-PCR MasterMix (bulk volume, 20 *μ*l). In the RT-PCR Master Mix SYBR-Green I dye fluorescence signal intensity was associated with the quantity of DNA and 55 point fluorescence was set-up as the monitoring point. The comparative Ct value method, using a housekeeping gene (GAPDH) as an internal standard, was employed to determine the relative levels of TGF-β1, α-SMA, N-cadherin, fibroblast-specific protein 1 (FSP1, also called S100A4), BMP-7 and E-cadherin.

### Western blotting

Whole cell proteins (20 *μ*g) were separated by PAGE and transferred to nylon membranes. The primary antibodies were as follows: anti-α-SMA (1:2,000 dilution; Dako, Carpinteria, CA, USA), anti-TGF-β1 (1:2,000 dilution; Santa Cruz Biotechnology Inc., Santa Cruz, CA, USA), anti-E-cadherin (1:2,000 dilution; USA), anti-BMP-7 (1:1,500 dilution; Cell Signaling Technology, Danvers, MA, USA), anti-FSP1 (1:2,000 dilution; Santa Cruz Biotechnology) and anti-GAPDH (1:2,000 dilution; Sigma-Aldrich). Appropriate secondary antibodies were used and antigens were detected by enhanced chemiluminescence (Pierce Biotechnology, Rockford, IL, USA).

### Statistical analyses

The SPSS 12.0 software (SPSS, Chicago, IL, USA) was used for statistical analysis. Quantitative variables of normality were tested and if the data conformed to a normal distribution they were expressed as means ± SD. Two independent t-tests were performed and if the data were not normally distributed, they were expressed as medians with a range and non-parametric tests were considered. For categorical data, non-parametric tests were used to compare frequencies. P<0.001 (two-sided) was considered to indicate statistically significant differences.

## Results

### Aspartate aminotransferase (AST) and alanine aminotransferase (ALT) detection

ALT and AST activities were significantly increased in the liver fibrosis model group compared with those in the normal control (P<0.001). In the alendronate sodium-treated group, the ALT and AST activities were markedly reduced compared with those in the liver fibrosis mice which were not treated with alendronate sodium (P<0.001).

### Alendronate sodium-treated pathology expressed in mouse liver fibrosis

**Figure 1:**
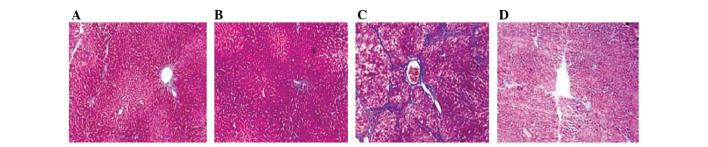
Hepatic tissue sections stained with Masson’s trichrome stain (×100). (A) Normal control group. (B) Alendronate sodium control group. (C) Liver fibrosis model group. (D) Alendronate sodium-treated group.

Following alendronate sodium (25 mg/kg) treatment twice each week for 8 weeks, hepatic tissue sections stained with Masson’s trichrome stain exhibited a reversal of mouse liver fibrosis to a significant extent (Fig. 1).

### Expression of α-SMA

**Figure 2:**
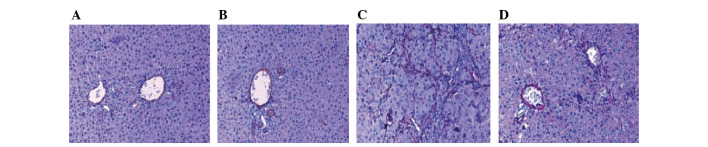
Alendronate sodium regulated expression of α-SMA in CCl_4_-induced hepatic fibrosis of rats (×200). (A) Normal control group. (B) Alendronate sodium control group. (C) Liver fibrosis model group. (D) Alendronate sodium-treated group. α-SMA, α-smooth muscle actin.

Following alendronate sodium treatment, the expression of α-SMA positive cells in the hepatic fibrosis area was significantly reduced (P<0.05; Fig. 2).

### Expression of TGF-β1, α-SMA, N-cadherin, FSP1, BMP-7 and E-cadherin mRNA

**Figure 3:**
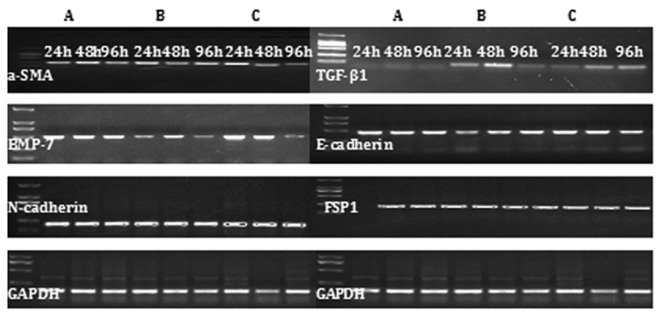
RT-PCR results demonstrating the effect of CCl_4_-induced mouse liver fibrosis on the expression of TGF-β1, α-SMA, N-cadherin, FSP1, BMP-7 and E-cadherin mRNA during EMT in (A) the control group, (B) the liver fibrosis model group and (C) the alendronate sodium-treated group during chronic exposure to TGF-β1 (5 ng/ml) from 24 to 96 h. TGF-β1, transforming growth factor-β1; α-SMA, α-smooth muscle actin; FSP1, fibroblast-specific protein 1; BMP-7, bone morphogenetic protein-7; EMT, epithelial-mesenchymal transition.

In the mouse liver fibrosis model, increased expression of TGF-β1, α-SMA, N-cadherin and FSP1 mRNA was observed, but the expression of of BMP-7 and E-cadherin was reduced. The opposite expression pattern was observed in the alendronate sodium-treated group (Fig. 3).

### Expression ratios of TGF-β1, α-SMA, BMP-7 and E-cadherin to GAPDH mRNA

**Figure 4:**
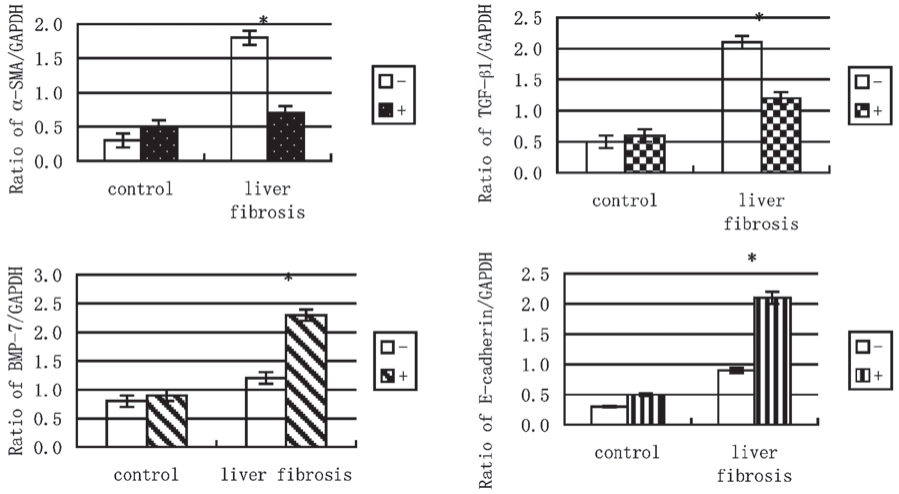
Ratio of α-SMA, TGF-β1, BMP-7 and E-cadherin to GAPDH in the alendronate sodium control and liver fibrosis model groups. (−) Primary mouse hepatocytes (1×10^6^/dish) were cultured for 48 h in F12 medium containing 10% fetal bovine serum and 5 *μ*g/ml insulin until they had adhered. (+) Primary mouse hepatocytes (1×10^6^/dish) were cultured for 48 h in F12 medium containing 10% fetal bovine serum, 5*μ*g/ml insulin and 50*μ*g/mL alendronate sodium. TGF-β1, transforming growth factor-β1; α-SMA, α-smooth muscle actin; BMP-7, bone morphogenetic protein-7.

Chronic exposure to TGF-β1 (5 ng/ml) for 96 h increased the expression ratios of α-SMA and TGF-β1 to GAPDH but following alendronate sodium treatment, the expression ratio of α-SMA to GAPDH decreased. The BMP-7 and E-cadherin to GAPDH ratios, however, were increased further by the alendronate sodium treatment (Fig. 4).

### Protein expression of E-cadherin and α-SMA in each group

**Figure 5:**
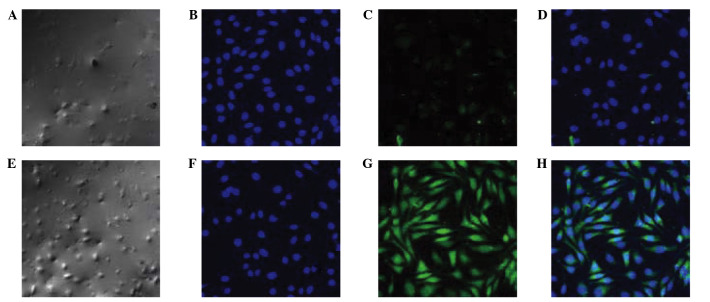
Protein expression of E-cadherin and α-SMA in mouse hepatic tissue (immunofluorescence method). (A–D) protein expression of E-cadherin; (E–H) protein expression of α-SMA; (A and E) alendronate sodium control group; (B,C,D,F,G and H) alendronate sodium-treated group; (B and F) protein expression of E-cadherin and α-SMA with green fluorescence; (C and G) protein expression of E-cadherin and α-SMA with blue fluorescence; (D and H) protein expression of E-cadherin and α-SMA with merged green and blue fluorescence. α-SMA, α-smooth muscle actin.

Following alendronate sodium treatment, the protein expression of E-cadherin (green fluorescence) was denser, while that of α-SMA (blue fluorescence) was more scattered than in the controls (Fig. 5).

### Analysis of E-cadherin in alendronate sodium-treated cells

**Figure 6:**
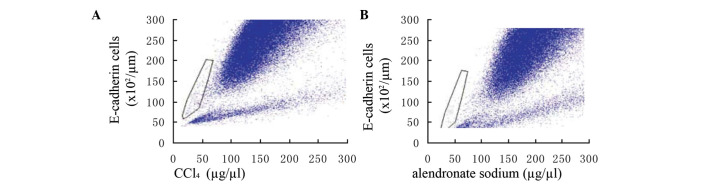
E-cadherin cell analysis by TGF-β1-induced EMT in CCl_4_-induced liver fibrosis of mice. (A) Liver fibrosis model group. (B) Alendronate sodium-treated group. The area highlighted by a polygon exhibit an average amplification efficiency of E-cadherin after 50 *μ*g/*μ*l CCl_4_ or alendronate sodium. Each E-cadherin cell assay is experimentally validated using dissociation (melt) curve analysis and agarose gel verification. After 50 *μ*g/*μ*l alendronate sodium, E-cadherin in the cells of the hepatic fibrosis area were greater and more dense in the alendronate sodium-treated group than the liver fibrosis model group. TGF-β1, transforming growth factor-β1, EMT, epithelial-mesenchymal transition.

Following alendronate sodium treatment, the expression of E-cadherin in the cells of the hepatic fibrosis area was greater and denser than in the controls. (Fig. 6)

## Discussion

The effects of TGF-β1-induced EMT on the structure, migration, cytoskeletal dynamics and long-term correlations of mammalian epithelial cell lines have been investigated with time-resolved impedance analysis (8). Liver sections were labeled to detect antigens associated with biliary epithelial cells (E-cadherin), EMT [FSP1, vimentin, N-cadherin, matrix metalloproteinase (MMP)-2 and α-SMA] and intracellular signal-transduction mediated by phosphorylated (p) Smad 2/3 (9).

The mechanisms underlying the morphological and phenotypic changes of epithelial markers undergoing EMT in liver fibrosis include a loss of E-cadherin and cytokeratin; increased expression of FSP1, vimentin, N-cadherin and α-SMA; basement membrane component loss; and the production of interstitial-type matrix molecules, including fibronectin and type I/III collagen (10).

In the present study, significantly less α-SMA and TGF-β1 and more BMP-7 and E-cadherin was expressed in the alendronate sodium-treated group and the controls than in the CCl_4_-induced mouse liver fibrosis model group (P<0.001). Following the exposure of these primary mouse hepatocytes to 5 ng/ml TGF-β1 for 96 h, the alendronate sodium-treated group exhibited increased staining for the epithelial markers E-cadherin which was accompanied by the decreased expression of various mesenchymal markers, including α-SMA and FSP-1. Alendronate sodium may also affect the activities of several signaling pathways that trigger the EMT, such as the Notch, Wnt and integrin pathways (11). The present study demonstrated that in the liver fibrosis model mice the expression of TGF-β1 and α-SMA was increased. However, in the alendronate sodium-treated group this was accompanied by the decreased expression of TGF-β1 and α-SMA, but increased the expression of BMP-7 and E-cadherin observed by western blotting.

The administration of alendronate, a potent inhibitor of bone resorption, was observed to be associated with an increase in the bone mineral density. Alendronate also reduced the activity of the parathyroid hormone which also stimulates bone resorption, thereby releasing preformed growth factors that are adsorbed to the bone matrix, such as insulin-like growth factor 1 and TGF-β1 (12). However, the role of alendronate in liver fibrosis remains unclear. Alendronate may regulate tissue remodeling by controlling TGF-β1-induced profibrotic functions in liver fibrosis. Thus the data of the present study suggest that EMT occurred in mouse liver fibrosis and induced the accumulation of TGF-β1-and α-SMA-expressing mesodermal cells while expanding the endodermal compartment during liver morphogenesis, suggesting that alendronate may also aid the reversal of mouse liver fibrosis (13).

EMT is induced by the integrated actions of numerous stimuli, including TGF-β1 and matrix-generated signals that are also known to be implicated in inflammation, repair responses and fibrosis (14). Chronic exposure to TGF-β1 induces the transition of hepatocytes to collagen-producing mesenchymal cells and the prolonged exposure of hepatocytes to TGF-β1 increases the expression of collagen and induces cytoskeletal rearrangement that resembles the EMT (15). These morphological and molecular alterations may provide the foundation for liver fibrosis.

Consideration of the association and mechanisms of EMT and alendronate in liver fibrosis suggests that the underlying mechanism is associated with changes in the redox state and that alendronate markedly decreased the expression of TGF-β1 and α-SMA in the liver tissue. The redox state may remain variable in liver fibrosis and depends on the balance between TGF-β/smad- and BMP-7-modulated mechanisms that regulate EMT and mesenchymal-epithelial transition (MET) in multifunctional progenitors. In all these processes, TGF-β1 acts profibrogenically while alendronate has opposing effects. The balance of these cytokines is further modulated by TGF-β1 which reduces alendronate activities. This may explain the mechanism of hepatic fibrosis. EMT may be important for the diagnosis of hepatic fibrosis and for developing studies of the pathogenesis of hepatic fibrosis and establishing effective preventive approaches.

The present study revealed that alendronate reduces the ability of TGF-β1 to increase the induction of EMT in liver fibrosis which was consistent with the hypothesis that TGF-β1 signaling induces the EMT through various signaling mechanisms and is the predominant agent mediating these fibrotic changes. Alendronate was identified following previous descriptions characterising its biological activity in extracts of demineralised bone. Alendronate also synthesises a number of growth regulatory peptides which are stored in the bone matrix and are possibly responsible for normal bone formation.

## Abbreviations:

TGF-β1: transforming growth factor-β1;
BMP-7: bone morphogenetic protein-7;
EMT: epithelial-mesenchymal transition;
MET: mesenchymal-epithelial transition;
α-SMA: α-smooth muscle actin
